# The Prognostic Value of Left Atrial Size and Strain Assessed by Cardiac Magnetic Resonance in the Coronary Chronic Total Occlusion

**DOI:** 10.3390/jcdd13030111

**Published:** 2026-02-27

**Authors:** Jinfan Tian, Wenxiao Xia, Xueyao Yang, Mingduo Zhang, Huijuan Zuo, Libo Liu, Min Zhang, Yuan Zhou, Ziyu An, Xin Zhao, Lijun Zhang, Yi He, Xiantao Song

**Affiliations:** 1 Department of Cardiology, Beijing Anzhen Hospital, Capital Medical University, Beijing 100029, China; tjfbama@mail.ccmu.edu.cn (J.T.); 2001071@mail.ccmu.edu.cn (W.X.); yangxueyao@mail.ccmu.edu.cn (X.Y.); zhangmingduo@ccmu.edu.cn (M.Z.); liboliu2007@mail.ccmu.edu.cn (L.L.); minjun1977@sina.com (M.Z.); zhouyuanfw1019@sina.com (Y.Z.); anziyu@mail.ccmu.edu.cn (Z.A.); zhaoxin_0825@mail.ccmu.edu.cn (X.Z.); 2 Department of Community Health Research, Beijing Anzhen Hospital, Capital Medical University, Beijing 100050, China; huijuanzuo@126.com; 3 Department of Radiology, Beijing Anzhen Hospital, Capital Medical University, Beijing 100029, China; 4 Department of Radiology, Beijing Friendship Hospital, Capital Medical University, Beijing 100050, China; heyi139@sina.com

**Keywords:** cardiac magnetic resonance, coronary occlusion, left atrial volume index, left atrial strain, prognosis

## Abstract

Background: The relationship between left atrial (LA) size, LA strain, and long-term prognosis in patients with coronary chronic total occlusion (CTO) remains unclear. This study aimed to evaluate the association of LA size and LA strain with clinical outcomes in CTO patients using cardiac magnetic resonance (CMR). Methods: This retrospective study included 168 patients with left ventricular ejection fraction (LVEF) ≥ 40%. The primary endpoint was the composite of major adverse cardiovascular and cerebrovascular events (MACCE). Model 1 was established by adjusting for clinically relevant parameters and standard CMR metrics. Models 2–4 were developed using Cox regression based on Model 1, with additional adjustment for each LA strain parameter separately. Results: A total of 168 patients with an LVEF ≥ 40% were analyzed, of whom 39 (23.2%) experienced MACCE during a mean follow-up of 45.9 months (median, 42 months). A preliminary model suggested that LA maximum volume index (LAVI_max_) was independently associated with MACCE (HR 1.05, 95% CI 1.02–1.08, *p* = 0.004). Specifically, compared to the first quartile of LAVI_max_, the second, third, and fourth quartiles were associated with an increased risk of MACCE (Q2: HR 4.50, 95% CI 1.42–14.27, *p* = 0.011; Q3: HR 4.40, 95% CI 1.29–14.96, *p* = 0.018; Q4: HR 5.55, 95% CI 1.71–18.06, *p* = 0.004). In Models 2–4, higher LAVI_max_ remained independently associated with MACCE (all *p* < 0.05), after adjusting for LA reservoir strain, conduit strain and booster strain, separately. In contrast, none of the LA strain parameters were associated with MACCE. Conclusions: Among CTO patients with LVEF ≥ 40%, LAVI_max_ was independently associated with MACCE.

## 1. Introduction

Coronary chronic total occlusion (CTO) presents a significant clinical challenge. The benefits of percutaneous coronary intervention (PCI) and/or coronary artery bypass grafting (CABG) for CTO patients remain uncertain [1,2,3]. Previous longitudinal studies evaluating CTO outcomes have utilized left ventricular ejection fraction (LVEF), myocardial viability, left ventricular (LV) strain, and/or routine clinical factors for risk prediction [2,3,4,5]. Additionally, left atrial (LA) strain has been demonstrated to predict adverse cardiovascular events [6,7,8]. However, the prognostic value of LA size and/or function in the context of CTO is still unclear. In several prior randomized CTO trials and a prospective registry, the reported mean LVEF was generally in the preserved range (≥50%), indicating that many enrolled CTO patients had preserved systolic function [1,2,3,4]. Cardiac magnetic resonance (CMR) is a unique imaging modality that can comprehensively characterize cardiac morphology, function, perfusion, tissue viability, and quality within a single examination. Fast LA long-axis strain (LA-LAS) analysis is an innovative technique that can assess LA size and function based on CMR scans [9]. Therefore, the aim of our study was to evaluate the association of LA size and LA strain with clinical outcomes in patients with a single CTO lesion and LVEF ≥ 40% within a retrospective CMR cohort.

## 2. Materials and Methods

### 2.1. Study Population

**Figure 1:**
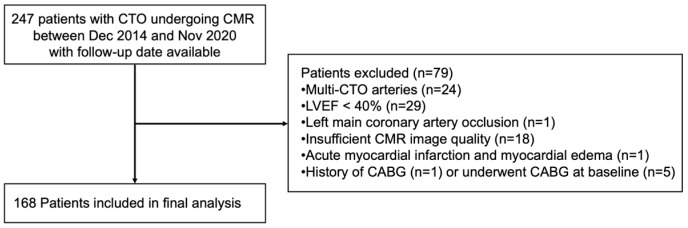
Study protocol.

We consecutively screened 247 patients with CTO who underwent CMR at our facility between December 2014 and November 2020, with follow-up data available. A total of 168 patients were included in our final analysis. The inclusion criteria were as follows: (1) age ≥ 18 years, (2) coronary angiography-confirmed CTO, and (3) LVEF ≥ 40%. The exclusion criteria were as follows: (1) patients with multi-CTO arteries (*n* = 24); (2) patients with LVEF < 40% (*n* = 29); (3) patients with left main coronary artery occlusion (*n* = 1); (4) patients with acute myocardial infarction and myocardial edema on CMR (*n* = 1); (5) patients with a history of CABG (*n* = 1) or who underwent CABG at baseline (n = 5); (6) patients with CMR images of insufficient quality for assessment (*n* = 18) (Figure 1).

We defined CTO as a lesion located in one of the three main epicardial arteries or a major side branch with a diameter greater than 2.5 mm. Multi-vessel disease (MVD) was defined as the presence of one CTO lesion co-occurring with ≥75% stenosis in one or more of the other arteries described above. Successful CTO revascularization was defined as restoration of antegrade flow in the target vessel with Thrombolysis in Myocardial Infarction (TIMI) flow grade ≥ 2, without in-hospital major procedural complications. In patients with MVD, revascularization strategies were individualized, and revascularization was performed based on clinical and procedural considerations. PCI could involve treatment of the CTO lesion alone or in combination with non-CTO lesions. The study was conducted in accordance with the Declaration of Helsinki (as revised in 2013). All patients provided written informed consent, and the experimental procedures and study protocols were approved by the ethics board of the Beijing Anzhen Hospital.

### 2.2. Definitions of Lesion and Clinical Endpoints

The primary endpoint was major adverse cardiovascular and cerebrovascular events (MACCE), defined as a composite of unplanned revascularization, non-fatal myocardial infarction (MI), in-stent restenosis, cardiac death, heart failure hospitalization, and stroke. All-cause death was not included as a separate endpoint, as no non-cardiovascular deaths were documented during follow-up.

### 2.3. CMR Protocol

All patients with CTO underwent CMR using a 3T scanner (MAGNETOM Verio, A Tim System; Siemens Healthineers, Erlangen, Germany; Achieva, Philips, Amsterdam, The Netherlands) equipped with a 32-element matrix coil. Cine imaging was conducted using contiguous short-axis slices that encompassed the entire LV and three long-axis views (2-, 3-, and 4-chamber) using a retrospective ECG-gated steady-state free precession sequence with 25 phases in the cardiac cycle (repetition time/echo time/flip angle = 1.5 ms/3.4 ms/50°, time resolution = 41 ms, slice thickness = 8 mm). Late gadolinium enhancement (LGE) imaging was performed at least 10 min following the intravenous administration of 0.2 mmol/kg gadolinium.

### 2.4. CMR Strain Analysis

**Figure 2:**
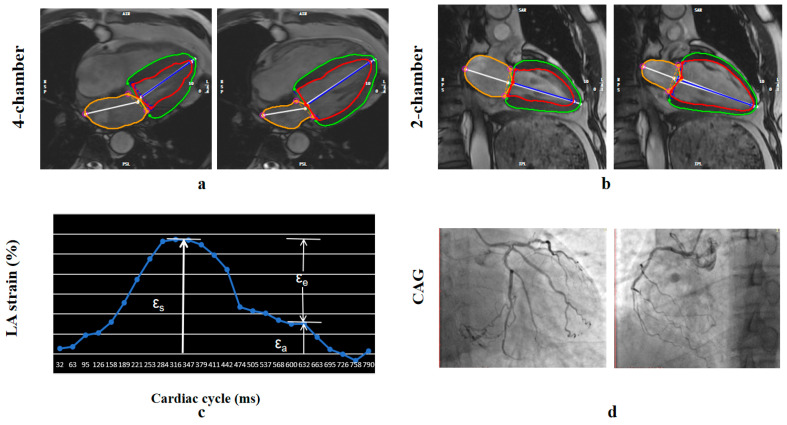
Fast semi-automated left atrial (LA) longitudinal strain. (**a**,**b**) LA tracking in cine CMR: (**a**) four- and (**b**) two-chamber views. (**c**) Squares denote the anatomic reference points (atrioventricular junction and mid posterior LA wall) that were tracked automatically throughout the cardiac cycle. The strain of each wall was calculated as a percentage using the strain formula. (**d**) Coronary angiography showing chronic total occlusion of the right coronary artery. ε_a_ = booster strain, ε_e_ = conduit strain, ε_s_ = reservoir strain. Red and green contours indicate the LV endocardial and epicardial borders, respectively; the orange contour indicates the LA border.

LA and LV myocardial strain measurements were conducted offline using cvi42 (Circle Cardiovascular Imaging, Inc., Calgary, AB, Canada). LA volume and function were assessed based on 2-chamber and 4-chamber long-axis views in the fast long-axis strain module of the software. Atrioventricular junctions and a user-defined point at the mid-posterior LA wall were selected on standard CMR 2- and 4-chamber views. Subsequently, the LA boundary of each image was automatically tracked, with manual adjustments made for optimal wall tracking (Figure 2). Curves depicting atrial volume and strain variations with cardiac cycle were obtained.

LA maximum volume index (LAVI_max_) was obtained at the frame with the maximum LA volume, and LA minimum volume index (LAVI_min_) was obtained at the frame with the minimum LA volume. These frames were software-defined as the time points corresponding to the maximum and minimum LA volumes, respectively. The LA emptying fractions were calculated from LA volume, and the LA volume index (LAVI) was derived by dividing LA volume by body surface area.

Three aspects of LA strain were analyzed, as illustrated in Figure 2, including total LA strain (ε_s_, regarding LA reservoir function), LA active strain (ε_a_, regarding LA booster pump), and LA passive strain (ε_e_, regarding LA conduit function).

**Figure 3:**
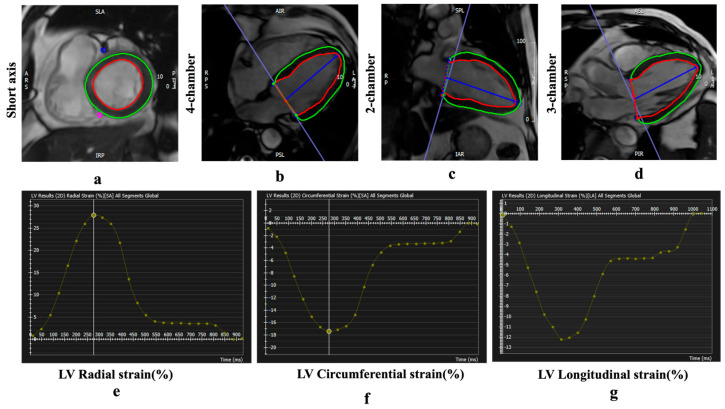
Examples of CMR feature tracking (FT) for quantification of myocardial deformation. (**a**–**d**) Cine CMR. Strain curves derived from FT analysis show peak global radial strain (GRS) (**e**), circumferential strain (GCS) (**f**), and longitudinal strain (GLS) values (**g**). Red and green contours indicate the LV endocardial and epicardial borders, respectively. Negative strain values (GCS and GLS) indicate myocardial shortening.

The endocardial and epicardial borders of the LV were traced using a threshold-based segmentation method at both end-diastole (as defined by the software) and end-systole (identified as the image with the smallest area of the left ventricle) in short- and long-axis cine images (2-chamber, 3-chamber, and 4-chamber). Trabeculations and papillary muscles were included in the ventricular volume assessment. LVEF and LVEDV were measured using the short-axis cine images in the short 3D module of the software, while LV strain was quantified using both short- and long-axis cine images in the tissue tracking module (Figure 3). Peak global radial strain (GRS) and global circumferential strain (GCS) were derived from 2D short-axis acquisitions, while peak global longitudinal strain (GLS) was obtained from 2D long-axis acquisitions.

### 2.5. CMR LGE Analysis

**Figure 4:**
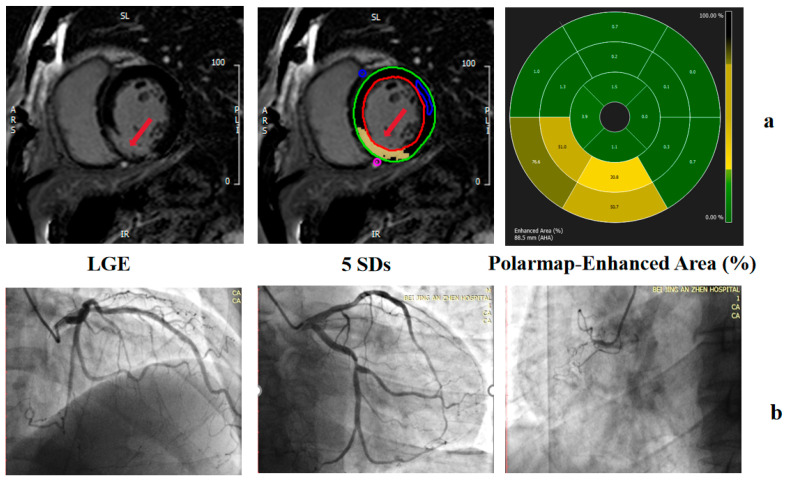
Examples of grayscale thresholding techniques on LGE images. LGE imaging before planimetry and thresholding techniques were performed. (**a**) EV measured by 5SD. (**b**) Coronary angiography showing chronic total occlusion of the right coronary artery. The red arrow indicates the infarcted myocardium on LGE images.

The LV endocardial and epicardial traces on the LGE images were performed manually to determine the myocardial mass, excluding the papillary muscles and intertrabecular blood pool from the total myocardial mass. Normal myocardium was defined visually as a region of myocardium without any apparent LGE on visual inspection. The mean signal intensity and standard deviation (SD) were determined by drawing a region of interest (ROI) in a portion of the normal myocardium (a sample of at least 100 pixels per ROI) on three consecutive midventricular image sections (Figure 4). The mean signal intensities and SDs were averaged across the three midventricular sections to yield an average mean signal intensity and SD. The presence and quantity of LGE were determined with grayscale thresholds of 5 SDs. The total myocardial mass (TMM), total myocardial volume (TMV) of the LV, total enhanced mass (TEM), total enhanced volume (TEV), and the proportion of TEV to TMV (EV%) in 5 SDs were recorded.

### 2.6. Reproducibility

Two experienced radiologists analyzed LV myocardial strain and the LGE images. To calculate inter-observer and intra-observer agreement, endocardial and epicardial borders were retraced across 20 patients and thresholds were redetermined. To assess intra-observer reproducibility, the same observer analyzed the same participants twice within a one-month interval. Inter-observer reproducibility was assessed with two observers who were blinded to each other’s findings.

### 2.7. Statistical Analysis

Continuous variables were assessed for normality and are presented as mean ± standard deviation for normally distributed data and as median (interquartile range) for non-normally distributed data. Categorical variables are presented as counts. Baseline characteristics were compared using independent sample *t*-tests for continuous variables and chi-square tests for categorical variables. Differences in LVEF, LVEDV, GLS, GCS, and left atrial ejection fraction (LAEF) between patients with and without MACCE were compared using independent sample *t*-tests. Left ventricular end-systolic volume (LVESV, mL), LAVI_max_(mL/m^2^), LAVI_min_ (mL/m^2^), LAV_max_/LVESV ratio, LV Mass (indexed, g/m^2^), GRS, LA reservoir strain, LA conduit strain, LA booster strain, and LGE parameters were compared between patients with and without MACCE using non-parametric tests. Correlations between CMR parameters were assessed using Pearson’s correlation coefficients. Intraclass correlation coefficients (ICCs) were used to measure inter- and intra-observer variability.

Risk predictors for MACCE were assessed using Cox regression models, which included key clinical characteristics related to MACCE and CMR indices. Model 1 incorporated the following variables: age, sex, hypertension, diabetes, hyperlipidemia, smoking status, drinking status, successful CTO-PCI, LAD occlusion, MVD and standard CMR indices (LVEF, EV, GCS, GLS, LAEF, LAVI_max_). Absolute values of GLS and GCS were included in all multivariable models. These clinical variables were consistently included in all subsequent multivariable models. Given the relatively small sample size and limited number of events, and because LV volume/function indices (e.g., LVEDV, LVESV, and LVEF) are closely related and clinically relevant, we adjusted for LV function using LVEF and did not additionally include LVEDV or LVESV in the multivariable Cox models. Three additional multivariable analyses were performed to evaluate the predictive value of LA reservoir strain (Model 2: Model 1 + reservoir strain), conduit strain (Model 3: Model 1 + conduit strain), and booster strain (Model 4: Model 1 + booster strain). In Models 1 through 4, LAVI_max_ was included either as a continuous variable or as a categorical variable based on quartiles (Q1: 0–25.121; Q2: 25.121–31.242; Q3: 31.242–38.429; Q4: >38.429). To investigate the relationship between LAV_max_/LVESV ratio and MACCE, univariate and multivariable Cox regression analyses were performed in the Supplementary Table S2, with covariates including important clinical characteristics, LVEF, EV, GCS, GLS, LAEF and LAV_max_/LVESV. Because the ratio LAV_max_/LVESV is derived from LAV_max_ and has a potential correlation with LAVI_max_, these measures were not entered simultaneously into the same multivariable model; therefore, LAVI_max_ was not taken into account in this model (Supplementary Table S2). Hazard ratios (HRs) and their corresponding 95% confidence intervals (CIs) were calculated using multivariable Cox proportional hazards models. A two-sided *p*-value < 0.05 was considered statistically significant. To investigate the shape of the association between LAVI_max_ and MACCE, a multivariable Cox proportional hazards model incorporating restricted cubic splines (RCS) was constructed, adjusted for age, sex, drinking status, hypertension, diabetes, hyperlipidemia, smoking status, LAD-CTO, CTO procedural success, MVD, and LAEF. Knots were placed at the 10th, 50th, and 90th percentiles of LAVI_max_, with the reference value (HR = 1) set at the 25th percentile. The number and location of knots were determined according to the minimum Akaike information criterion (AIC). SPSS (version 21, IBM, Armonk, NY, USA) and R (version 4.4.0) were used for data management and statistical analyses.

## 3. Results

### 3.1. Baseline Characteristics of the Study Population

A total of 247 patients with CTO who underwent CMR imaging at our facility between December 2014 and November 2020, with follow-up data available, were screened for inclusion in our study. From this cohort, 168 participants (56.88 ± 10.07 years, 81% male) were included in the final analysis (Figure 1). The main CTO target vessels were the right coronary artery (RCA) (*n* = 88, 52.4%) and the left anterior descending artery (LAD) (*n* = 59, 35.1%). 100 patients had single-vessel CTO without concomitant significant disease in other coronary arteries, while 68 patients had MVD. Among the 100 patients with single-vessel CTO without non-CTO lesion in other epicardial arteries, successful CTO revascularization was achieved in 66 patients, whereas 25 patients experienced unsuccessful CTO-PCI attempts and 9 patients did not undergo CTO intervention. Among the 68 patients with MVD, successful CTO revascularization was achieved in 54 patients. In most of these successful cases (51 patients), CTO-PCI was performed in combination with PCI of non-CTO lesions, while only 3 patients underwent CTO-only intervention. The remaining 14 patients with MVD either experienced unsuccessful CTO-PCI attempts or did not undergo CTO intervention. CMR was performed within one month of the procedure in most patients.

During a mean follow-up of 45.9 months (median, 42 months) from the baseline CMR examination, 39 of the 168 patients (23.2%) experienced MACCE, and 19 patients were censored during follow-up due to loss to follow-up. These MACCEs included one case of cardiac death, one case of acute non-fatal myocardial infarction requiring unplanned revascularization, 12 cases of unplanned revascularization due to in-stent restenosis, 19 cases of unplanned revascularization due to non–in-stent restenosis, five cases of in-stent restenosis without subsequent unplanned revascularization, and one case of stroke. The prevalence of hypertension, diabetes mellitus, smoking, drinking, history of percutaneous coronary intervention, and medications were similar between patients who did and did not experience MACCE (*p* > 0.05) (see Table 1). Only one patient received MRA therapy, and no patients were treated with SGLT2 inhibitors.

### 3.2. CMR Baseline Characteristics

Baseline CMR characteristics stratified by MACCE status are shown in Table 2. Compared with patients without MACCE, those with MACCE had larger LV end-diastolic and end-systolic volumes, lower LVEF, and higher indexed LV mass (all *p* < 0.05). No significant between-group differences were observed in GLS, GRS, or GCS.

Among LA parameters, LAVI_min_ was higher and LA reservoir strain was significantly lower in the MACCE group (both *p* < 0.05). No significant differences were observed in LA conduit strain, booster strain, LGE extent, LAVI_max_ or the LAV_max_/LVESV ratio.

### 3.3. Associations Between LAVI_max_ and MACCE

A higher LAVI_max_ (continuous) was found to be independently associated with MACCE based on Model 1, which incorporates clinically relevant parameters and standard CMR indices (HR 1.05, 95% CI 1.02–1.08, *p* = 0.004) (see Table 3 and Supplementary Table S1). Patients with hyperlipidemia and MVD were more likely to experience MACCE (refer to Supplementary Table S1). In comparison to the Q1 of LAVI_max_, patients in Q2, Q3, and Q4 exhibited a significantly higher risk of MACCE (Q2: HR 4.50, 95% CI 1.42–14.27, *p* = 0.011; Q3: HR 4.40, 95% CI 1.29–14.96, *p* = 0.018; Q4: HR 5.55, 95% CI 1.71–18.06, *p* = 0.004) (see Table 3). In contrast, when LAV_max_/LVESV was included in univariate and multivariable Cox regression models (Supplementary Table S2), no significant association with MACCE was observed. This suggests that absolute LA enlargement, rather than its proportion relative to LV size, may be more closely associated with adverse cardiovascular outcomes in this cohort.

LA reservoir strain, conduit strain, and booster strain were individually incorporated into multivariable analysis in Model 2, Model 3, and Model 4. These models also included LAVI_max_, either as a continuous variable or categorized into quartiles. Additionally, factors including male sex, age, smoking and drinking status, hypertension, diabetes, hyperlipidemia, CTO vessel (LAD), successful CTO-PCI, MVD, LVEF, GCS, GLS, LAEF, and EV were taken into account in Models 2, 3, and 4 (as detailed in Table 4 and Supplementary Table S3).

From Model 2 to Model 4, a higher LAVI_max_ (continuous) was independently associated with MACCE (Model 2: HR 1.05, 95% CI 1.01–1.08, *p* = 0.007; Model 3: HR 1.05, 95% CI 1.02–1.08, *p* = 0.005; Model 4: HR 1.05, 95% CI 1.02–1.09, *p* = 0.004) when adjusting for LA reservoir strain, conduit strain and booster strain separately. In contrast, none of the LA strain parameters (reservoir, conduit, or booster) were associated with the outcome (Supplementary Table S3 and Table 4).

Compared to LAVI_max_ quartile Q1, quartiles Q2–Q4 were consistently associated with a higher risk of MACCE across Models 2–4. In Model 2, the hazard ratios (HRs) were 4.41 (95% CI 1.37–14.15, *p* = 0.013) for Q2, 4.33 (95% CI 1.26–14.83, *p* = 0.020) for Q3, and 5.42 (95% CI 1.64–17.91, *p* = 0.006) for Q4. In Model 3, the corresponding HRs were 4.35 (95% CI 1.37–13.83, *p* = 0.013) for Q2, 4.35 (95% CI 1.27–14.90, *p* = 0.019) for Q3, and 5.61 (95% CI 1.72–18.34, *p* = 0.004) for Q4. In Model 4, HRs were 4.61 (95% CI 1.45–14.68, *p* = 0.010) for Q2, 4.51 (95% CI 1.32–15.42, *p* = 0.016) for Q3, and 5.82 (95% CI 1.76–19.26, *p* = 0.004) for Q4 (Table 4).

**Figure 5:**
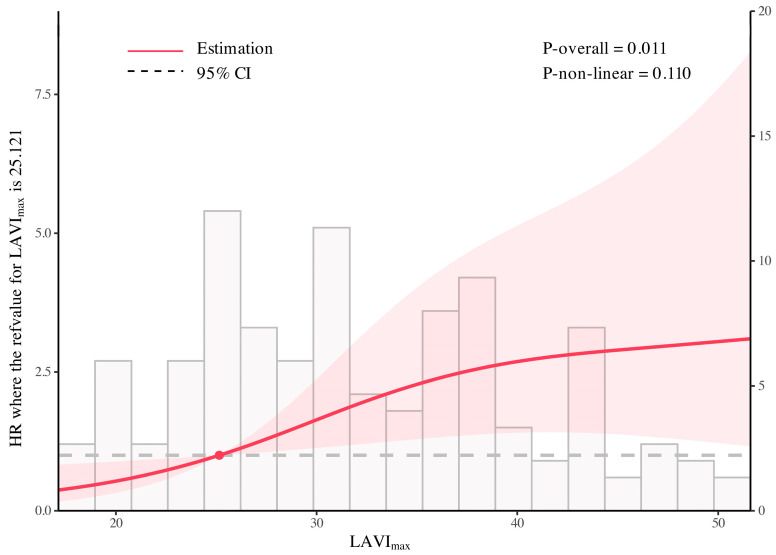
Association between LAVI_max_ and adverse outcomes analyzed using a multivariable Cox model with restricted cubic splines. The multivariable Cox model with restricted cubic splines showed a significant overall association between LAVI_max_ and adverse outcomes (P-overall = 0.011), whereas the non-linear component was not statistically significant (P-non-linear = 0.110). The solid line indicates the estimated HR, the dot marks the reference value, and the shaded zone represents the 95% CI.

The multivariable Cox model with RCS showed a significant overall association between LAVI_max_ and adverse outcomes (P-overall = 0.011), whereas the non-linear component was not statistically significant (P-non-linear = 0.110). Using the 25th percentile of LAVI_max_ (25.121 mL/m^2^) as the reference (HR = 1), higher LAVI_max_ values were associated with a higher estimated hazard ratio compared with the reference level. This overall association was consistent with the trend observed in the multivariable Cox regression models using LAVI_max_ stratification (Figure 5).

### 3.4. Intra- and Inter-Observer Agreement

Intra-observer reproducibility ranged from good to excellent between the first and second evaluations for the three LA strain parameters (ICC = 0.83–0.96, *p* < 0.01). Inter-observer reproducibility also ranged between good and excellent between the observers for the three LA global strain parameters (ICC = 0.80–0.95, *p* < 0.01). Intra-observer reproducibility ranged between good and excellent when the first and second evaluations for the three LV strain parameters were compared (ICC = 0.79–0.95, *p* < 0.01). Inter-observer reproducibility was good between the two observers for the three LV global strain parameters (ICC = 0.77–0.89, *p* < 0.01).

## 4. Discussion

Here, we found that, for patients with CTO and LVEF ≥ 40%, LAVI_max_ was independently associated with MACCE. This study provides new evidence for the prognostic assessment of CTO patients with LVEF ≥ 40%. The main finding of this study is that, among patients with CTO and LVEF ≥ 40%, left atrial enlargement was independently associated with clinical outcomes. The relationship between LA strain and MACCE in the CTO cohort with LVEF ≥ 40% requires further investigation.

Patients with LA enlargement tended to have increased atrial intraluminal stiffness, elevated LV end-diastolic pressure, worsening LV diastolic dysfunction, and impaired reservoir function, which have been linked to adverse prognosis [10]. LA enlargement has been previously identified as an independent risk factor for MACCE among patients with acute coronary syndrome (ACS) [11], atrial fibrillation undergoing catheter ablation [12], hypertrophic cardiomyopathy [13] and/or type 2 diabetes mellitus [14]. A meta-analysis of 2705 ACS patients from 11 cohort studies with a mean follow-up time of 18.7 ± 9.8 months showed that patients with low LAVI had a decreased risk of major adverse cardiac events (15.9% vs. 33.7%), decreased long-term all-cause mortality (9.14% vs. 18.1%), decreased short-term mortality (3.31% vs. 9.38%), and lower hospitalization rates (11.6% vs. 25.5%) compared to patients with increased LAVI [15]. Similarly, Liu et al. [16] reported that higher LAVI, whether analyzed continuously or by quartiles, was associated with increased MACCE (composite of all-cause death, MI, and stroke) in patients with three-vessel coronary disease after a median follow-up of 6.6 years. These findings suggest that LA enlargement, as a marker of chronic pressure and volume overload, may serve as a surrogate for longstanding cardiac remodeling processes that are not fully captured by LVEF alone. However, it remains unclear whether LA enlargement increases MACCE risk in CTO patients, especially among those with preserved LVEF. There are several potential explanations as to why increased LAVI_max_ is associated with adverse outcomes. First, patients with LA enlargement may have decreased coronary flow reserve compared to those with normal LA sizes [17,18]. Second, patients with LA enlargement also commonly have comorbid hypertension, which may increase MACCE risk [16]. Third, LA remodeling may also reflect chronic neurohormonal activation, including upregulation of the renin–angiotensin–aldosterone system and sympathetic nervous system [10]. These systemic changes can exacerbate myocardial fibrosis, oxidative stress, and inflammation, thereby contributing to cardiovascular events on multiple levels. These three factors may collectively contribute to the association between higher LAVI_max_ and increased MACCE risk observed in our study.

In addition, LA enlargement increases the risk of atrial fibrillation, which is associated with a higher risk of stroke and heart failure. However, in our cohort with LVEF ≥ 40%, no cases of new-onset atrial fibrillation or heart failure hospitalization were observed during follow-up. LA strain is associated with MACCE in patients who have heart failure with reduced LVEF [19]. However, in our cohort, we did not find that LA strain had prognostic value for MACCE incidence.

This finding should be interpreted in the context of both technical and population-related factors. Previous studies have shown that, compared with healthy individuals, LV strain of CTO patients was impaired [20]. However, specific CMR-based reference ranges for LA strain in patients with CTO are currently lacking, particularly when stratified by preserved versus reduced LVEF. Although the temporal resolution of routine clinical CMR is suitable for standard functional assessment, absolute values of CMR-derived atrial strain are influenced by technical characteristics of cine imaging, which should be taken into account when evaluating strain-based parameters. Importantly, in patients with CTO and LVEF ≥ 40%, left atrial functional changes likely reflect long-term adaptive remodeling. Although LA strain may be reduced compared with healthy reference populations [21], these changes appear to remain within an adaptive range and do not reach a threshold that confers incremental prognostic information. As a result, LA strain fails to establish a clear risk gradient for adverse outcomes in this population, which may explain its lack of prognostic value in the present study. This differs from acute myocardial infarction, in which cardiac function is markedly impaired and LA strain is closely associated with prognosis [22].

It should be noted that although LAVI_max_ analyzed as a continuous variable was independently associated with MACCE, the magnitude of the effect estimate was modest (HR 1.05 per 1 mL/m^2^ increase). This finding may reflect the gradual and cumulative nature of LA remodeling, whereby small incremental increases in atrial volume may not translate into an immediately apparent clinical risk, but may become clinically relevant across a broader range of values. Notably, patients in the present cohort had LVEF ≥ 40%, which may have limited the ability to detect a stepwise increase in MACCE risk with further LA enlargement. Consistently, when LAVI_max_ was analyzed by quartiles, patients in Q2–Q4 exhibited a substantially higher risk of MACCE compared with Q1, while the hazard ratios across Q2–Q4 remained relatively similar. This pattern may indicate a relatively similar risk level across higher LAVI_max_ categories within the current quartile-based categorization in this cohort. Similar trends were observed across Models 2–4, supporting the consistency of this observation. Furthermore, the wide confidence intervals observed in the RCS analysis (Figure 5) are likely attributable to the limited sample size and the relatively small number of events, particularly at the extremes of LAVI_max_ distribution. Therefore, these spline-based analyses should be interpreted as exploratory and hypothesis-generating rather than as definitive tools for individual risk prediction, although the overall direction of the association remains consistent with the categorical and continuous Cox regression analyses.

Our Cox regression model showed that CTO-PCI did not impact MACCE incidence, which is consistent with most current randomized clinical trials. This reinforces previous evidence suggesting that while CTO-PCI may improve symptoms and quality of life, it does not consistently reduce hard clinical endpoints such as MACCE. CMR characteristics may, therefore, provide important prognostic information before successful CTO-PCI. In this context, multimodal cardiovascular imaging may be increasingly important for cardiovascular prevention, complementing traditional risk stratification and imaging metrics. Techniques such as echocardiography, cardiac magnetic resonance, and computed tomography provide structural, functional, and tissue characterization, enabling early detection of subclinical disease and more precise risk stratification. The integration of these modalities can guide tailored preventive strategies and enhance prognostic assessment in at-risk populations [23]. CMR-based feature-tracking strain has methodological limitations compared with echocardiography (e.g., longer acquisition time, relatively lower temporal resolution, and potential contouring-related variability), and intervendor differences in post-processing software may further affect measurement consistency. In addition, CMR-derived strain values may not be directly interchangeable with echocardiographic measurements, which may influence cross-modality generalizability; moreover, the higher cost and limited availability of CMR in some settings may restrict the generalizability and clinical applicability of these findings.

This study has several limitations. First, this was a single-center retrospective cohort study with a relatively small sample size. A larger study with longer follow-up may provide more rigorous evidence. Second, in the retrospective CMR-based CTO cohort reviewed at our center, patients with overt heart failure were limited in number; therefore, the present analysis primarily included patients with LVEF ≥ 40%, which may limit generalizability to populations with more advanced systolic dysfunction. Third, revascularization strategies and procedural success were not uniform across the study population, particularly in patients with MVD, reflecting real-world clinical practice; therefore, residual confounding related to interventional strategy cannot be excluded. Finally, as this was a retrospective study, clinical events were identified through medical record review and follow-up interviews and adjudicated by the investigators’ team, and attrition bias related to loss to follow-up cannot be excluded; moreover, detailed longitudinal data on treatment adherence and risk factor control were not systematically available, and residual confounding related to variations in medical management remains possible; however, guideline-directed medical therapy was applied according to standard practice and patients received discharge counseling on risk factor control.

## 5. Conclusions

In conclusion, we found that among patients with a single CTO lesion and LVEF ≥ 40%, LA enlargement was associated with a higher risk for MACCE. This suggests an association between atrial volume parameters and clinical outcomes alongside clinical characteristics.

## Figures and Tables

**Table 1:** Baseline Characteristics of Participants with and Without MACCE.

Index	All Participants (*n* = 168)	MACCE (*n* = 39)	Non-MACCE (*n* = 129)	*p* Value
Age	56.88 ± 10.07	56.72 ± 10.44	56.92 ± 9.99	0.912
Sex (*n*, %)				0.506
Male	136 (81)	33 (84.6)	103 (79.8)	
Female	32 (19)	6 (15.4)	26 (20.2)	
Hypertension (*n*, %)	107 (63.7)	22 (56.4)	85 (65.9)	0.281
Diabetes (*n*, %)	50 (29.8)	12 (30.8)	38 (29.5)	0.875
Hyperlipidemia (*n*, %)	79 (47)	22 (56.4)	57 (44.2)	0.180
Kidney dysfunction (*n*, %)	4 (2.4)	2 (5.1)	2 (1.6)	0.230
Multi-vessel disease (*n*, %)	68 (40.5)	21 (53.8)	47 (36.4)	0.052
PCI history (*n*, %)	43 (25.6)	13 (33.3)	30 (23.3)	0.206
MI history (*n*, %)	29 (17.3)	7 (17.9)	22(17.1)	0.897
Smoking (*n*, %)	97 (57.7)	25 (64.1)	72 (55.8)	0.359
Drinking (*n*, %)	52 (31)	14 (35.9)	38 (29.5)	0.446
CTO-vessel (*n*, %)				0.467
LAD	59 (35.1)	12 (30.8)	47 (36.4)	
LCX	21 (12.5)	7 (17.9)	14 (10.9)	
RCA	88 (52.4)	20 (51.3)	68 (52.7)	
ACEI/ARB/ARNI (*n*, %)	80 (47.6)	19 (48.7)	61 (47.3)	0.875
β-blocker (*n*, %)	131 (78)	34 (87.2)	97 (75.2)	0.113

Abbreviations: MACCE, major adverse cardiovascular and cerebrovascular events; PCI, percutaneous coronary intervention; MI, myocardial infarction; CTO, coronary chronic total occlusion; LAD, left anterior descending artery; LCX, left circumflex artery; RCA, right coronary artery; ACEI, angiotensin-converting enzyme inhibitor; ARB, angiotensin II receptor blocker; ARNI, angiotensin receptor-neprilysin inhibitor.

**Table 2:** CMR Parameters of Participants with and Without MACCE.

Index	All Participants (*n* = 168)	MACCE (*n* = 39)	Non-MACCE (*n* = 129)	*p* Value
Functional parameters				
Left ventricle				
End-diastolic volume, mL	126.72 ± 30.71	135.75 ± 31.75	124.00 ± 29.99	0.036 *
End-systolic volume, mL	54.08 (43.48, 69.73)	62.26 (49.38, 77.88)	52.68 (40.73, 64.93)	0.006 **
Ejection fraction, %	55.65 ± 7.83	52.81 ± 7.01	56.51 ± 7.89	0.009 **
Mass, indexed, g/m^2^	50.76 (45.62, 58.01)	56.82 (49.57, 62.40)	49.99 (44.99, 56.63)	0.006 **
GLS, %	−14.61 ± 2.34	−14.35 ± 2.57	−14.68 ± 2.27	0.439
GRS, %	28.11 (23.53, 32.63)	26.30 (21.89, 31.30)	28.62 (24.29, 32.75)	0.064
GCS, %	−17.30 ± 2.73	−16.58 ± 2.82	−17.52 ± 2.68	0.060
Left atrium				
LAVI_min_, index, mL/m^2^	14.05 (10.93, 17.24)	14.94 (12.38, 18.66)	13.52 (10.50, 16.87)	0.045 *
LAVI_max_, index, mL/m^2^	31.24 (25.12, 38.43)	32.27 (26.94, 41.51)	30.93 (24.43, 38.22)	0.120
Ejection fraction, %	54.96 ± 7.55	53.54 ± 6.55	55.39 ± 7.79	0.178
Reservoir strain, %	26.48 (21.55, 33.02)	25.11 (19.41, 30.08)	27.60 (22.09, 33.76)	0.046 *
Conduit strain, %	11.01 (7.50, 15.60)	9.72 (5.93, 16.75)	11.48 (7.85, 15.55)	0.201
Booster strain, %	15.17 (11.80, 19.10)	14.25 (10.55, 19.05)	15.98 (12.59, 19.12)	0.162
EV				
LGE extent (% of LV volume) (*n* = 167)	6.48 (2.13, 15.03)	8.03 (2.85, 17.39)	5.95 (2.09, 14.60)	0.236
LAV_max_/LVESV	1.09 (0.86, 1.38)	1.06 (0.84, 1.27)	1.12 (0.86, 1.46)	0.264

Abbreviations: LAVI_min_, left atrial minimum volume index; LAVI_max_, left atrial maximum volume index; GCS, global circumferential strain; GRS, global radial strain; GLS, global longitudinal strain; EV, total enhanced volume/myocardial volume; LGE, late gadolinium enhancement. LVESV, LAVI_max_ (mL/m^2^), LAVI_min_ (mL/m^2^), LAV_max_/LVESV, LV Mass (indexed, g/m^2^), GRS, LA reservoir strain, LA conduit strain, LA booster strain, and LGE parameters are presented as median (interquartile range). * *p* value < 0.05; ** *p* value < 0.01.

**Table 3:** Univariate and Multivariable Cox Proportional Hazards Model for MACCE Using Important Baseline Characteristics and Standard CMR indexes (Model 1).

	Univariable Analysis			Multivariable Analysis (Model 1)		
	Unadjusted HR	95% CI	*p* value	Adjusted HR	95% CI	*p* value
LAVI_max_ (continuous)	1.03	1.01–1.06	0.019 *	1.05	1.02–1.08	0.004 **
LAVI_max_ (Q1)	Reference			Reference		
LAVI_max_ (Q2)	2.90	1.02–8.25	0.046 *	4.50	1.42–14.27	0.011 *
LAVI_max_ (Q3)	2.72	0.93–8.00	0.068	4.40	1.29–14.96	0.018 *
LAVI_max_ (Q4)	3.68	1.29–10.51	0.015 *	5.55	1.71–18.06	0.004 **

Abbreviations: MACCE, major adverse cardiovascular and cerebrovascular events; LAVI_max_, left atrial maximum volume index. * *p* value < 0.05; ** *p* value < 0.01.

**Table 4:** Multivariable Cox Proportional Hazards Model for MACCE Risk Using Important Clinical Variables and CMR indexes, with Separate Adjustment for LA Reservoir Strain, LA Conduit Strain and LA Booster Strain (Models 2–4).

	Model 2 Multivariable Analysis with LA Reservoir Strain			Model 3 Multivariable Analysis with LA Conduit Strain			Model 4 Multivariable Analysis with LA Booster Strain		
	Adjusted HR	95% CI	*p* value	Adjusted HR	95% CI	*p* value	Adjusted HR	95% CI	*p* value
LAVI_max_ (continuous)	1.05	1.01–1.08	0.007 *	1.05	1.02–1.08	0.005 **	1.05	1.02–1.09	0.004 **
LAVI_max_ (Q1)	Reference			Reference			Reference		
LAVI_max_ (Q2)	4.41	1.37–14.15	0.013 *	4.35	1.37–13.83	0.013 *	4.61	1.45–14.68	0.010 *
LAVI_max_ (Q3)	4.33	1.26–14.83	0.020 *	4.35	1.27–14.90	0.019 *	4.51	1.32–15.42	0.016 *
LAVI_max_ (Q4)	5.42	1.64–17.91	0.006 **	5.61	1.72–18.34	0.004 **	5.82	1.76–19.26	0.004 **

Abbreviations: MACCE, major adverse cardiovascular and cerebrovascular events; LAVI_max_, left atrial maximum volume index. **, *p* < 0.01; *, *p* < 0.05. Models 2–4 were built on Model 1 by additionally including LA reservoir strain, LA conduit strain, and LA booster strain, respectively.

## Data Availability

The data presented in this study are available on request from the corresponding author due to patient privacy and ethical restrictions.

## Supplementary Materials

The following supporting information can be downloaded at: https://www.mdpi.com/article/10.3390/jcdd13030111/s1. Supplementary Table S1. Univariate and multivariable Cox Proportional Hazards Model for MACCE Using Important Baseline Characteristics and left atrial volume index at end-diastole; Supplementary Table S2. Univariate and multivariable Cox Proportional Hazards Model for MACCE Using Important Baseline Characteristics and LAVmax/LVESV; Supplementary Table S3. Multivariable Cox Proportional Hazards Model for MACCE Risk by Using Important Clinical variables, LA Reservoir strain, LA Conduit strain and LA Booster strain.

## Abbreviations

The following abbreviations are used in this manuscript:

CTO	coronary chronic total occlusion
LV	left ventricular
LA	left atrial
CMR	cardiac magnetic resonance
LVEF	left ventricular ejection fraction
EV	total enhanced volume/myocardial volume
LAEF	left atrial ejection fraction
LGE	late gadolinium enhancement
LA-LAS	LA long-axis strain
MVD	multi-vessel disease
MACCE	major adverse cardiovascular and cerebrovascular events
MI	myocardial infarction
LAVI_max_	left atrial maximum volume index.
LAVI_min_	left atrial minimum volume index
LAVI	LA volume index
LVEDV	left ventricular end-diastolic volume
LVESV	left ventricular end-systolic volume
GRS	global radial strain
GCS	global circumferential strain
GLS	global longitudinal strain
TMM	total myocardial mass
TMV	LV total myocardial volume
TEM	total enhanced mass
TEV	total enhanced volume
ICCs	intraclass correlation coefficients
RCA	right coronary artery
LAD	left anterior descending artery
PCI	percutaneous coronary intervention

## References

[1] Werner G.S., Martin-Yuste V., Hildick-Smith D., Boudou N., Sianos G., Gelev V., Rumoroso J.R., Erglis A., Christiansen E.H., Escaned J. (2018). A randomized multicentre trial to compare revascularization with optimal medical therapy for the treatment of chronic total coronary occlusions. Eur. Heart J..

[2] Werner G.S., Hildick-Smith D., Yuste V.M., Boudou N., Sianos G., Gelev V., Rumoroso J.R., Erglis A., Christiansen E.H., Escaned J. (2023). Three-year outcomes of A Randomized Multicentre Trial Comparing Revascularization and Optimal Medical Therapy for Chronic Total Coronary Occlusions (EuroCTO). EuroIntervention.

[3] Lee S.W., Lee P.H., Ahn J.M., Park D.W., Yun S.C., Han S., Kang H., Kang S.J., Kim Y.H., Lee C.W. (2019). Randomized Trial Evaluating Percutaneous Coronary Intervention for the Treatment of Chronic Total Occlusion. Circulation.

[4] Hirai T., Grantham J.A., Sapontis J., Cohen D.J., Marso S.P., Lombardi W., Karmpaliotis D., Moses J., Nicholson W.J., Pershad A. (2019). Quality of life changes after chronic total occlusion angioplasty in patients with baseline refractory angina. Circ. Cardiovasc. Interv..

[5] Elias J., Van Dongen I.M., Hoebers L.P., Ouweneel D.M., Claessen B.E., Råmunddal T., Laanmets P., Eriksen E., Piek J.J., Van der Schaaf R.J. (2020). Recovery and prognostic value of myocardial strain in ST-segment elevation myocardial infarction patients with a concurrent chronic total occlusion. Eur. Radiol..

[6] Thomas L., Muraru D., Popescu B.A., Sitges M., Rosca M., Pedrizzetti G., Henein M.Y., Donal E., Badano L.P. (2020). Evaluation of left atrial size and function: Relevance for clinical practice. J. Am. Soc. Echocardiogr..

[7] Hoit B.D. (2014). Left atrial size and function: Role in prognosis. J. Am. Coll. Cardiol..

[8] Gan G.C., Kadappu K.K., Bhat A., Fernandez F., Gu K.H., Cai L., Byth K., Eshoo S., Thomas L. (2021). Left atrial strain is the best predictor of adverse cardiovascular outcomes in patients with chronic kidney disease. J. Am. Soc. Echocardiogr..

[9] Leng S., Ge H., He J., Kong L., Yang Y., Yan F., Xiu J., Shan P., Zhao S., Tan R.S. (2020). Long-term Prognostic Value of Cardiac MRI Left Atrial Strain in ST-Segment Elevation Myocardial Infarction. Radiology.

[10] Thomas L., Abhayaratna W.P. (2017). Left atrial reverse remodeling: Mechanisms, evaluation, and clinical significance. JACC Cardiovasc. Imaging.

[11] Ri T., Saito C., Arashi H., Yamaguchi J., Ogawa H., Hagiwara N. (2022). Increased left atrial volume index is associated with more cardiovascular events in patients with acute coronary syndrome: HIJ-PROPER study findings. Echocardiography.

[12] Ishiguchi H., Yoshiga Y., Shimizu A., Fukuda M., Omuro A., Hisaoka M., Nakashima Y., Fujita M., Hashimoto S., Omuro T. (2024). Novel Method for Risk Stratification of Major Adverse Clinical Events Using Pre- and Post-Ablation Left Atrial Volume Index in Patients with Persistent Atrial Fibrillation. Circ. Rep..

[13] Tani T., Yagi T., Kitai T., Kim K., Nakamura H., Konda T., Fujii Y., Kawai J., Kobori A., Ehara N. (2011). Left atrial volume predicts adverse cardiac and cerebrovascular events in patients with hypertrophic cardiomyopathy. Cardiovasc. Ultrasound.

[14] Poulsen M.K., Dahl J.S., Henriksen J.E., Hey T.M., Høilund-Carlsen P.F., Beck-Nielsen H., Møller J.E. (2013). Left atrial volume index: Relation to long-term clinical outcome in type 2 diabetes. J. Am. Coll. Cardiol..

[15] Ahmeti A., Bytyçi F.S., Bielecka-Dabrowa A., Bytyçi I., Henein M.Y. (2021). Prognostic value of left atrial volume index in acute coronary syndrome: A systematic review and meta-analysis. Clin. Physiol. Funct. Imaging.

[16] Liu R., Song L., Zhang C., Jiang L., Tian J., Xu L., Feng X., Wan L., Zhao X., Xu O. (2024). Implications of left atrial volume index in patients with three-vessel coronary disease: A 6.6-year follow-up cohort study. Chin. Med. J..

[17] Koh A.S., Murthy V.L., Sitek A., Gayed P., Bruyere J., Wu J., Di Carli M.F., Dorbala S. (2015). Left atrial enlargement increases the risk of major adverse cardiac events independent of coronary vasodilator capacity. Eur. J. Nucl. Med. Mol. Imaging.

[18] Galderisi M., Cicala S., Caso P., De Simone L., D’Errico A., Petrocelli A., de Divitiis O. (2002). Coronary flow reserve and myocardial diastolic dysfunction in arterial hypertension. Am. J. Cardiol..

[19] Sengeløv M., Jørgensen P.G., Jensen J.S., Bruun N.E., Olsen F.J., Fritz-Hansen T., Nochioka K., Biering-Sørensen T. (2015). Global Longitudinal Strain Is a Superior Predictor of All-Cause Mortality in Heart Failure with Reduced Ejection Fraction. JACC Cardiovasc. Imaging.

[20] Zhang L., Tian J., Yang X., Liu J., He Y., Song X. (2022). Quantification of strain analysis and late gadolinium enhancement in coronary chronic total occlusion: A cardiovascular magnetic resonance imaging follow-up study. Quant. Imaging Med. Surg..

[21] Truong V.T., Palmer C., Wolking S., Sheets B., Young M., Ngo T.N.M., Taylor M., Nagueh S.F., Zareba K.M., Raman S. (2020). Normal left atrial strain and strain rate using cardiac magnetic resonance feature tracking in healthy volunteers. Eur. Heart J. Cardiovasc. Imaging.

[22] Zhang M., Li Z., Wang Y., Chen L., Ren Y., Wu Y., Wang J., Lu Y. (2024). Left atrial and ventricular longitudinal strain by cardiac magnetic resonance feature tracking improves prognostic stratification of patients with ST-segment elevation myocardial infarction. Int. J. Cardiovasc. Imaging.

[23] Carerj M.L., Restelli D., Poleggi C., Di Bella G., Zito C., Manganaro R., Piccione M.C., Trimarchi G., Farina A., Micari A. (2025). The Role of Imaging in Cardiovascular Prevention: A Comprehensive Review. J. Cardiovasc. Echogr..

