# Supplementary material for: The Prognostic Value of Left Atrial Size and Strain Assessed by Cardiac Magnetic Resonance in the Coronary Chronic Total Occlusion
Source: J Cardiovasc Dev Dis. 2026 Feb 27;13(3):111. doi: 10.3390/jcdd13030111 (PMC13026679; doi:10.3390/jcdd13030111)
Supplement: Supplementary file 1 [file jcdd-13-00111-s001.zip › jcdd-4041245-supplementary.pdf]

Supplementary Table S1. Univariate and multivariable Cox Proportional Hazards Model for MACCE Using Important Baseline Characteristics and CMR indexes ($LAVI_{max}$).

	Univariable analysis			Multivariable analysis (Model 1)		
	Unadjusted HR	95% CI	*p* value	Adjusted HR	95% CI	*p*-value
Male	1.40	0.59–3.35	0.445	1.96	0.60–6.43	0.268
Age	1.00	0.97–1.03	0.976	1.02	0.98–1.06	0.382
LVEF	0.94	0.91–0.98	0.006**	0.97	0.91–1.03	0.340
EV	1.02	0.99–1.05	0.270	1.00	0.97–1.04	0.811
GCS	0.88	0.78–0.99	0.030*	0.93	0.75–1.15	0.507
GLS	0.89	0.77–1.03	0.108	1.10	0.90–1.34	0.340
Smoking	1.26	0.65-2.42	0.495	0.99	0.44–2.23	0.975
Drinking	1.39	0.72–2.67	0.328	0.95	0.42–2.12	0.893
Hypertension	0.83	0.44–1.56	0.560	0.55	0.27–1.14	0.109
Diabetes	1.07	0.54–2.11	0.850	1.36	0.66–2.81	0.403

Hyperlipidemia	1.69	0.89–3.20	0.111	2.44	1.08–5.49	0.031*
Multi-vessel disease	3.35	1.71–6.57	<0.001***	3.79	1.74–8.25	0.001**
CTO vessel (LAD)	0.76	0.38–1.50	0.424	0.91	0.42–1.97	0.802
Successful CTO-PCI	1.21	0.59–2.48	0.603	0.90	0.41–1.96	0.786
$LAVI_{max}$ (continuous)	1.03	1.01–1.06	0.019*	1.05	1.02–1.08	0.004**
LA Ejection fraction	0.97	0.93–1.00	0.103	0.99	0.94–1.04	0.645

Abbreviations: MACCE, major adverse cardiovascular and cerebrovascular events; LVEF, left ventricular ejection fraction; EV, total enhanced volume / myocardial volume; GCS, global circumferential strain; GLS, global longitudinal strain; CTO, coronary chronic total occlusion; LAD, left anterior descending artery; PCI, percutaneous coronary intervention; $LAVI_{max}$, left atrial maximum volume index; LA, left atrial. Absolute values of GLS and GCS were included into all multivariable models. **p* value<0.05; ***p* value<0.01; ****p* value<0.001.

Supplementary Table S2. Univariate and multivariable Cox Proportional Hazards Model for MACCE Using Important Baseline Characteristics and CMR indexes (LAV_{max}/LVESV).

	Univariable analysis	Multivariable analysis

	Unadjusted HR	95% CI	*p* value	Adjusted HR	95% CI	*p* value
Male	1.40	0.59–3.35	0.445	2.06	0.63–6.71	0.232
Age	1.00	0.97–1.03	0.976	1.02	0.98–1.06	0.451
LVEF	0.94	0.91–0.98	0.006**	0.94	0.87–1.01	0.074
EV	1.02	0.99–1.05	0.270	1.01	0.97–1.05	0.591
GCS	0.88	0.78–0.99	0.030*	0.95	0.77–1.17	0.625
GLS	0.89	0.77–1.03	0.108	1.10	0.90–1.34	0.342
Smoking	1.26	0.65-2.42	0.495	0.83	0.36–1.90	0.653
Drinking	1.39	0.72–2.67	0.328	1.24	0.58–2.66	0.575
Hypertension	0.83	0.44–1.56	0.560	0.59	0.28–1.24	0.159
Diabetes	1.07	0.54–2.11	0.850	1.31	0.63–2.70	0.468
Hyperlipidemia	1.69	0.89–3.20	0.111	1.94	0.90–4.16	0.090
Multi-vessel disease	3.35	1.71–6.57	<0.001***	4.07	1.85–8.93	<0.001***
CTO vessel (LAD)	0.76	0.38–1.50	0.424	0.92	0.43–1.98	0.838
Successful CTO-PCI	1.21	0.59–2.48	0.603	0.91	0.42–1.97	0.806

LAV_{max}/LVESV	0.64	0.29–1.44	0.284	2.96	0.80–10.97	0.104
LA Ejection fraction	0.97	0.93–1.00	0.103	0.99	0.94–1.03	0.565

Abbreviations: MACCE, major adverse cardiovascular and cerebrovascular events; LVEF, left ventricular ejection fraction; EV, total enhanced volume / myocardial volume; GCS, global circumferential strain; GLS, global longitudinal strain; CTO, coronary chronic total occlusion; LAD, left anterior descending artery; PCI, percutaneous coronary intervention; $LAVI_{max}$, left atrial maximum volume index; LA, left atrial; LVESV, left ventricular end-systolic volume. **p* value<0.05; ***p* value<0.01; ****p* value<0.001.

Supplementary Table S3. Multivariable Cox Proportional Hazards Model for MACCE Risk Using Important Clinical Variables and CMR indexes, with Separate Adjustment for LA Reservoir Strain, LA Conduit Strain and LA Booster Strain (Models 2–4).

	Model 2 Multivariable Analysis with LA Reservoir Strain			Model 3 Multivariable Analysis with LA Conduit Strain			Model 4 Multivariable Analysis with LA Booster Strain		
	Adjusted HR	95% CI	*p* value	Adjusted HR	95% CI	*p* value	Adjusted HR	95% CI	*p* value

Male	2.02	0.61-6.66	0.251	2.08	0.62-6.90	0.234	1.92	0.58-6.38	0.285
Age	1.02	0.98-1.06	0.375	1.01	0.97-1.05	0.518	1.02	0.98-1.05	0.448
LVEF	0.97	0.91-1.03	0.316	0.97	0.91-1.03	0.314	0.97	0.91-1.04	0.379
EV	1.00	0.97-1.04	0.834	1.00	0.97-1.04	0.834	1.01	0.97-1.04	0.788
GCS	0.93	0.75-1.16	0.535	0.93	0.75-1.16	0.537	0.93	0.75-1.15	0.485
GLS	1.10	0.91-1.34	0.336	1.10	0.90-1.33	0.369	1.10	0.90-1.34	0.356
Smoking	0.98	0.43-2.22	0.963	1.02	0.45-2.31	0.960	1.01	0.44-2.31	0.977
Drinking	0.94	0.42-2.10	0.883	0.91	0.41-2.04	0.826	0.94	0.42-2.10	0.882
Hypertension	0.56	0.27-1.16	0.119	0.58	0.28-1.20	0.143	0.55	0.27-1.15	0.110
Diabetes	1.36	0.66-2.80	0.406	1.35	0.66-2.78	0.416	1.36	0.66-2.81	0.402
Hyperlipidemia	2.43	1.08-5.48	0.032*	2.40	1.06-5.44	0.036*	2.44	1.08-5.49	0.032*
Multi-vessel disease	3.69	1.68-8.10	0.001**	3.79	1.76-8.21	0.001**	3.94	1.76-8.81	0.001**

CTO vessel (LAD)	0.90	0.42-1.96	0.793	0.87	0.40-1.90	0.719	0.89	0.41-1.95	0.778
Successful CTO-PCI	0.92	0.42-2.02	0.832	0.91	0.42-2.00	0.821	0.88	0.40-1.93	0.743
$LAVI_{max}$ (continuous)	1.05	1.01-1.08	0.007**	1.05	1.02-1.08	0.005**	1.05	1.02-1.09	0.004**
LA Ejection fraction	1.00	0.94-1.06	0.909	1.00	0.95-1.05	0.937	0.98	0.93-1.04	0.549
LA Reservoir strain	0.99	0.94-1.04	0.717	NA	NA	NA	NA	NA	NA
LA Conduit strain	NA	NA	NA	0.97	0.90-1.04	0.360	NA	NA	NA
LA Booster strain	NA	NA	NA	NA	NA	NA	1.01	0.95-1.08	0.694

Abbreviations: MACCE, major adverse cardiovascular and cerebrovascular events; LA, left atrial; LVEF, left ventricular ejection fraction; EV, total enhanced volume / myocardial volume; GCS, global circumferential strain; GLS, global longitudinal strain; CTO, coronary chronic total occlusion; LAD, left anterior descending artery; PCI, percutaneous coronary intervention; $LAVI_{max}$, left atrial maximum volume index. *p value<0.05; **p value<0.01.
